# Conflict in Somalia: impact on child undernutrition

**DOI:** 10.1136/bmjgh-2016-000262

**Published:** 2017-05-29

**Authors:** Damaris K Kinyoki, Grainne M Moloney, Olalekan A Uthman, Ngianga-Bakwin Kandala, Elijah O Odundo, Abdisalan M Noor, James A Berkley

**Affiliations:** 1 INFORM Project, Spatial Health Metrics Group, Kenya Medical Research Institute/Wellcome Trust Research Programme, Nairobi, Kenya; 2 Nutrition Section, United Nations Children's Fund (UNICEF), Kenya Country Office, UN Complex Gigiri, Nairobi, Kenya; 3 Warwick Medical School, Health Sciences Research Institute, University of Warwick, Warwick Evidence, Gibbet Hill, Coventry, UK; 4 Department of Mathematics and Information sciences, Faculty of Engineering and Environment, Northumbria University, Newcastle upon Tyne, UK; 5 Faculty of Health and Sport Sciences, University of Agder, Kristiansand, Norway; 6 Division of Epidemiology and Biostatistics, School of Public Health, University of the Witwatersrand, Johannesburg, South Africa; 7 Food Security and Nutrition Analysis Unit (FSNAU) - Somalia, Food and Agriculture Organization of the United Nations, Ngecha Road Campus, Nairobi, Kenya; 8 Centre for Tropical Medicine and Global Health, Nuffield Department of Clinical Medicine, University of Oxford, CCVTM, Oxford, UK; 9 Kenya Medical Research Institute/Wellcome Trust Research Programme, Centre for Geographic Medicine Research (Coast), Kilifi, Kenya

**Keywords:** Undernutrition, wasting, stunting, conflict, Somalia, spatial-temporal modelling

## Abstract

**Introduction:**

In Somalia, protracted conflict and drought have caused population displacement and livelihood destruction. There is also widespread childhood undernutrition. We aimed to determine the independent effects of conflict on wasting and stunting among children aged 6–59 months nationwide in Somalia.

**Methods:**

Data were from household surveys during 2007–2010, including 73 778 children in 1066 clusters, the Armed Conflict Location and Event Data project database and remote sensing. We used Bayesian hierarchical spatial-temporal regression to examine the effects of conflict on wasting and stunting. Models included individual, household and environmental covariates and recent (<3 months) or longer term (3–12 months) conflict events.

**Results:**

15 355 (21%) and 22 739 (31%) observations were from wasted and stunted children, respectively. The conflict was associated with undernutrition independently of the individual, household and environmental factors, and its inclusion improved model performance. Recent conflict was associated with wasting (OR 1.37, 95% credible interval (CrI): (1.33, 1.42) and attributable fraction (AF) 7.6%)) and stunting (OR 1.21, 95% CrI (1.15, 1.28), AF 6.9%). Longer term conflict had greater effects on wasting (OR 1.76, 95% CrI (1.71, 1.81), AF 6.0%) and stunting (OR 1.88, 95% CrI = (1.83, 1.94), AF 7.4%). After controlling for conflict, the harmful effect of internal displacement and protective effects of rainfall and vegetation cover on undernutrition were enhanced.

**Conclusion:**

Conflict and internal displacement have large effects on undernutrition in ways not fully captured by simply measuring individual, household and environmental factors or drought.

**Key questions**

**What is already known about this topic?**

- Conflict negatively impacts children’s nutritional status in many low-income and middle-income countries.
- In Somalia, the effects of conflict have been compounded by concurrent drought and other long-term extrinsic environmental factors that predispose children to undernutrition. However, the effects of conflict have not been quantified anywhere in relation to individual, household, drought and other environmental determinants.

**What are the new findings?**

- To our knowledge, this is the first study to undertake a nationwide investigation of the effect of recent and longer term conflicts on child nutritional status controlling for other determinants.
- The effects of recent and longer term conflict, after adjustment for the individual, household and environmental determinants, were large, with a 76% and 88% greater odds of wasting and stunting and nationally, attributable fractions of 13% and 14%, respectively.

**Recommendations for policy**

- Inclusion of internally displaced persons, seasonality and details of conflict events at a cluster level are especially important in survey design and guiding response.

## Introduction

Conflict disrupts food production, increases food insecurity and causes population movement, including into internally displaced person (IDP) camps.^1 2^ Infectious diseases, exacerbated by the destruction of health facilities, hardship and hunger have direct effects on health and may also reduce human capacity for food production.^3^ In Somalia, the effects of conflict have been compounded by concurrent drought and other long-term extrinsic environmental factors that are predisposed to undernutrition, especially among children and pregnant women.^3^

The impact of conflict on the nutritional status of a population depends on the nature of the conflict, its duration, how it affects livelihoods and the coping strategies adopted by the community.^4^ The full impact may only be observed months or years after a specific conflict episode.^5^ Previous studies in sub-Saharan Africa indicate that, compared with preconflict periods, recent conflict is associated with high rates of undernutrition and under-5 mortality, and increased risk of low birth weight.^6 7^ Nutrition-specific interventions have been adopted in such settings as standard components of the humanitarian response in complex emergencies.^8^ Although the humanitarian response is commonly focused on the acute situation, it can have longer term positive impact in reducing undernutrition in the population.^2^

In Somalia, conflict has been cited as the main impediment to accessing healthcare and humanitarian aid.^9^ In the South Central zone, where there has been frequent conflict, undernutrition in children under 5 years old is reported to be at critical levels.^10^ In this zone, settlement patterns in both urban and agricultural areas have undergone substantial changes, with large influxes of non-resident clans, supported by their militias for economic gains. This has disrupted agro-pastoral livelihoods.^4^ In the north of Somalia, in areas enjoying relative stability and development, undernutrition rates are substantially lower.^11^ Substantial pockets of high vulnerability also occur in IDP camps and urban areas in the country.^11^

The majority of the Somali population depend on subsistence farming and pastoralism for their livelihood, and hence on the climate.^12^ Failure of successive rains, and consequently drought, across Somalia, has rendered this population vulnerable, especially in the populated southern region.^13^ As a result, communities have fought over natural resources, resulting in losses of vegetation cover. For example, in 2004, during the peak of the 4-year drought, the pastoralist community resorted to cutting and burning wood to make charcoal as a source of income.^13^

Previous analyses of the determinants of undernutrition in Somalia have not included conflict,^14^ which is likely to be key drivers of undernutrition. We, therefore, aimed to determine the impact of conflict events on wasting and stunting among children aged 6–59 months, adjusting for child, household and community characteristics, climate and environmental factors. We integrated anthropometric and household data from nutrition surveys conducted by the Food Security and Nutrition Analysis Unit (FSNAU) with data on conflict events from the Armed Conflict Location and Event Data (ACLED) project and environmental and climatic data from remote sensing.

## Methods

### Data and surveys

#### Nutritional survey data

Data were obtained from the FSNAU surveys of children aged 6–59 months between 2007 and 2010. Surveys were conducted biannually using stratified multistage cluster sampling design. The survey methods are described in detail elsewhere.^10^ At the individual child level, age, gender, weight, height, mid-upper arm circumference, vitamin A supplementation in the last 6 months, diarrhoea, acute respiratory infections (ARI) and febrile illness in the 2 weeks before the survey, and polio and measles vaccination history were collected. At the household level, information recorded included the household size and age structure, gender of the household head and access to different types of foods in the last 24 hours. Z-scores were computed for wasting and stunting using the WHO 2006 references.^15^ Wasting or stunting were defined as below −2 z-score for weight-for-height and height-for-age, respectively.

#### Conflict data

Information on conflict events was obtained from the ACLED database.^16^ The ACLED project was designed to capture a range of violent and non-violent actions by political agents including governments, rebel groups, militias, communal groups, political parties, rioters, protesters and civilians.^17^ It covers events occurring both within and outside the immediate context of the civil war, including violence against civilians, militia interactions, communal conflict and rioting.^16^ The data sources for this project included news reports, NGO and governmental agency publications, security alerts and published texts and books at international, regional and national levels. The project covers all countries in Africa from 1997 to 2015.^10^ ACLED reports specify nine types of conflict events: battle – no change of territory; battle – non-state actor overtakes territory; battle – government regains territory; headquarters or base established; strategic development; riots or protests; violence against civilians; non-violent transfer of territory; and remote violence.^17^ These event types were grouped into four classes for analysis: battle, remote violence, violence against civilians and others. A detailed description of conflict and definition of each category can be found in supplementary information file (online SI. 1 and Table SI. 1). These data were geocoded by event location and classified as occurring <3 months and 3–12 months before each nutritional survey to reflect the recent and longer term conflict for any conflict and each conflict event type.

10.1136/bmjgh-2016-000262.supp1 Supplementary data

#### Environmental data

A set of five distal environmental covariates associated with food security^18^ and vector-borne diseases^19^ on indices of undernutrition were examined: rainfall, enhanced vegetation index (EVI), mean temperature, distance to water and urbanisation. Rainfall and mean temperature were derived from the monthly average grid surfaces obtained from WorldClim database.^20^ The EVI values were derived from the MODerate-resolution Imaging Spectroradiometer sensor imagery^21^ for the period 2000–2010, while the urbanisation data was obtained from Global Rural Urban Mapping Project.^22^ All the environmental covariates were extracted from 1×1 km spatial resolution grids. Rainfall, temperature and EVI were summarised to compute seasonal averages using the four main seasons in Somalia: December–March: a harsh dry season called ‘Jilal’; April–June, which is the main rainy season called ‘Gu’; from July to September is the second dry season, the ‘Hagaa’; and October–December, the short rainy season known as ‘Deyr’.

## Ethical approval

Ethical approval was provided through permission by the Ministry of Health Somalia, Transitional Federal Government of Somalia Republic, Ref: MOH/WC/XA/146/07, dated 02/02/2007. Due to the high illiteracy rate of the population, informed consent was obtained verbally from all participating households and individuals.

## Statistical methods

Bayesian hierarchical spatial-temporal regression methods were used to quantify the effect of conflict on wasting and stunting. The household survey and environmental predictors of undernutrition were controlled at individual, household and community levels. Cluster level effects were incorporated into the model to allow for structured (spatial and temporal) and unstructured heterogeneity of undernutrition, using a convolution prior. District random effects were also included in this model to account for the variability between districts. Seasonality was controlled in the model as a factor with four unordered levels (December–March, April–June, July–September and October–December). Livelihood was also controlled as five unordered levels: agro-pastoral, pastoral, riverine, urban areas and IDPs.

As a first step, the associations of child-level, household and environmental covariates with wasting and stunting were examined in model A, not accounting for the effects of conflict. Then, conflict events were divided into two categories: (1) recent conflict: events within 3 months; and (2) longer term conflict: 3–12 months before each nutrition survey in model B, including the above covariates and conflict. Interaction effects between food and nutrition, environmental and conflict variables were also examined. All these models were implemented using Integrated Nested Laplace Approximation (INLA) as implemented in R-INLA in R project V.3.2.3.^23^ Detailed description of methods can be found in SI. 2 section in the supplementary information file.

The adjusted attributable fraction (AF) for recent and for longer term conflicts and EVI were computed as discussed by Rückinger *et al*.^24^ In this approach, the AFs are directly derived from logistic regression after removing the risk factors sequentially from the model by considering all permutations of the factors.^25 26^ The adjusted number of expected cases is determined from the predicted probabilities and then the AF is computed by subtracting the expected cases from the observed cases and by dividing by the observed cases.^24 26^ The resulting sequential AFs are sensitive to the order of removal of the risk factors in the model.^24 25^ This is addressed by computing the average of all obtained AFs.^24 25^ Using this approach, we determined the AFs of recent and longer term conflict and the EVI from the Bayesian spatial-temporal logistic regression by sequentially removing the three risk factors. The AFs from the all six possible permutations for the three factors were computed to get the final adjusted AFs of both wasting and stunting. The predicted probabilities for each individual were computed in a Bayesian framework only accounting for the explained variation in the model. This was done by multiplying the predicted probabilities by the proportion of the explained variation for each individual in the model.

To assess the predictive performance of the models, we used Watanabe-Akaike information criterion (WAIC) as implemented by Gelman and colleagues.^27^ This method estimates pointwise out-of-sample prediction accuracy from a fitted Bayesian model using the log-likelihood evaluated at the posterior simulations of the parameter values. WAIC is based on the series expansion of leave-one-out cross-validation.^27^ Furthermore, the observed and fitted prevalence of wasting and stunting from the models were compared using scatterplots.

## Results

Data from a total of 73 778 observations on children from 1066 clusters were examined (table 1). The mean age was 33 months (range: 6–59 months) and 47% were female. By livelihood zones, 42%, 27% and 16% of children were from areas of agro-pastoral, pastoral and riverine livelihoods, respectively, while 11% lived in IDP camps and 4% lived in urban areas. Overall, 15 355 (21%) and 22 739 (31%) of the children were wasted and stunted, respectively. Other characteristics of the children measured during the surveys are shown in Table SI. 2.

**Table 1:** A summary of the survey data used in this study by zone and region in Somalia. Children with weight-for-height and height-for-age z-scores of <-2 were considered stunted according to the WHO growth standards^15^

Zone	Region	Number of clusters	Number of children examined	Children stunted (%)	Children wasted (%)	Number of conflict events	Battle	Remote violence	Violence against civilians	Other	Total
North East (Puntland)	Bari	9	756	201 (26.59)	174 (23.02)	47	16	7	21	3	**47**
	Mudug	61	6188	804 (12.99)	1055 (17.05)	41	14	3	21	3	**41**
	Nugaal	24	1673	383 (22.89)	322 (19.25)	17	6	2	8	1	**17**
North West (Somaliland)	Awdal	26	862	7 (0.81)	177 (20.53)	7	2	0	5	0	**7**
	Sanaag	14	412	3 (0.73)	97 (23.54)	10	4	0	6	0	**10**
	Sool	3	142	18 (12.68)	24 (16.90)	27	8	0	11	8	**27**
	Togdheer	12	673	362 (53.79)	124 (18.42)	18	9	0	8	1	**18**
	Woqooyi Galbeed	23	2465	1378 (55.90)	480 (19.47)	27	5	0	19	3	**27**
South-Central	Bakool	75	3534	1150 (32.54)	1330 (37.63)	20	7	1	4	8	**20**
	Banadir	1	51	0 (0.00)	11 (21.57)	594	366	74	123	31	**594**
	Bay	98	5568	2133 (38.31)	1798 (32.29)	74	31	0	29	14	**74**
	Galgaduud	77	5831	1908 (32.72)	879 (15.07)	55	36	2	11	6	**55**
	Gedo	111	6985	1999 (28.62)	2616 (37.45)	31	11	0	7	13	**31**
	Hiraan	142	10 743	2260 (21.04)	2085 (19.41)	94	48	3	33	10	**94**
	Juba Dhexe	77	5253	2734 (52.05)	960 (18.28)	14	9	0	4	1	**14**
	Juba Hoose	71	5560	1553 (27.93)	926 (16.65)	60	26	1	20	13	**60**
	Shabelle Dhexe	101	7650	2414 (31.56)	1322 (17.28)	49	22	0	16	11	**49**
	Shabelle Hoose	141	9432	3432 (36.39)	1355 (14.37)	210	140	16	47	7	**210**
**Total**	**18**	**1066**	73 778	**22 739 (31%)**	**15 735 (21)**	**1395**	**760**	**109**	**393**	**133**	**1395**

### Model A: not accounting for the effect of conflict

As shown in table 2, pastoral communities had a lower risk of undernutrition than riverine communities. IDP communities were at a higher risk of both forms of undernutrition compared with agro-pastoral communities: OR=1.37, 95% credible interval (CrI): 1.34, 1.41 for wasting and OR=1.91, 95% CrI: 1.05, 3.48 for stunting.

**Table 2:** Multivariate adjusted odds ratio (AOR) and 95% credible interval (CrI) of the effect of covariates on wasting and stunting among children aged 6–59 months in Somalia. The estimates were derived from two models: model A that did not account for the effect of conflict as covariate model B that accounted for the effect of recent and longer term conflict. Values in bold typeface are those that do not contain the value 1 in their 95% CrI and were considered statistically significant

Correlates		Wasting				Stunting			
		Model A not controlling for the effect of conflict		Model B controlling for the effect of recent and longer term conflict		Model A not controlling for the effect of conflict		Model B controlling for the effect of recent and longer term conflict	
		OR	CrI	OR	CrI	OR	CrI	OR	CrI
**Conflict data**									
Recent conflict (within 3 months)				**1.37**	**(1.33 to 1.42)**			**1.21**	**(1.15 to 1.28)**
Longer term conflict (3–12 months ago)				**1.76**	**(1.71 to 1.81)**			**1.88**	**(1.83 to 1.94)**
**Livelihood** (agro-pastoral as reference)	Pastoral	**0.94**	**(0.91 to 0.97)**	**0.93**	**(0.91 to 0.96)**	**0.60**	**(0.41 to 0.87)**	**0.60**	**(0.41 to 0.87)**
	Riverine	**1.13**	**(1.09 to 1.17)**	**1.17**	**(1.13 to 1.21)**	**1.44**	**(1.04 to 1.99)**	**1.50**	**(1.45 to 1.55)**
	Urban	0.98	(0.94 to 1.02)	0.98	(0.93 to 1.04)	0.98	(0.93 to 1.04)	**0.83**	**(0.79 to 0.88)**
	IDP	**1.37**	**(1.34 to 1.41)**	**1.62**	**(1.54 to 1.70)**	**1.91**	**(1.05 to 3.48)**	**2.23**	**(2.07 to 2.40)**
**Child data**									
Vitamin A supplementation		**0.82**	**(0.81 to 0.84)**	**0.89**	**(0.87 to 0.91)**	1.10	(0.92 to 1.31)	0.90	(0.76 to 1.08)
Measles vaccination		0.87	(0.71 to 1.05)	**0.86**	**(0.84 to 0.88)**	0.87	(0.73 to 1.04)	**0.88**	**(0.84 to 0.91)**
Polio vaccination		**0.89**	**(0.87 to 0.91)**	**0.96**	**(0.94 to 0.98)**	**0.91**	**(0.87 to 0.94)**	**0.95**	**(0.93 to 0.97)**
Diarrhoea		**1.38**	**(1.16 to 1.64)**	**1.37**	**(1.15 to 1.63)**	**1.43**	**(1.21 to 1.68)**	**1.42**	**(1.34 to 1.50)**
Acute respiratory infection		**1.27**	**(1.07 to 1.50)**	**1.27**	**(1.07 to 1.50)**	**1.12**	**(1.08 to 1.16)**	**1.13**	**(1.09 to 1.17)**
Febrile Illness		**1.29**	**(1.08 to 1.53)**	**1.28**	**(1.08 to 1.52)**	**1.16**	**(1.12 to 1.20)**	**1.16**	**(1.12 to 1.21)**
Suspected measles		1.13	(0.98 to 1.32)	1.13	(0.98 to 1.31)	1.02	(0.99 to 1.04)	1.02	(0.78 to 1.34)
Sex of the child (female)		**0.72**	**(0.63 to 0.83)**	**0.72**	**(0.63 to 0.84)**	**0.75**	**(0.66 to 0.86)**	**0.75**	**(0.66 to 0.86)**
Chid age (<12 reference)	12–<24 months	**0.57**	**(0.53 to 0.61)**	**0.57**	**(0.44 to 0.74)**	**3.36**	**(3.27 to 3.46)**	**3.36**	**(2.53 to 4.47)**
	24–59 months	**0.69**	**(0.55 to 0.87)**	**0.69**	**(0.55 to 0.87)**	**2.34**	**(2.28 to 2.41)**	**2.34**	**(1.79 to 3.07)**
**Household data**									
Household size		**1.04**	**(1.01 to 1.08)**	**1.10**	**(1.06 to 1.13)**	**1.04**	**(1.01 to 1.08)**	**1.04**	**(1.00 to 1.07)**
Number of under-5		**1.07**	**(1.02 to 1.13)**	**1.03**	**(1.00 to 1.06)**	**1.04**	**(1.02 to 1.07)**	**1.03**	**(1.01 to 1.06)**
Female household head		1.06	(0.89 to 1.28)	1.08	(0.90 to 1.30)	**1.22**	**(1.02 to 1.45)**	**1.22**	**(1.02 to 1.46)**
Age of the mother		1.00	(0.99 to 1.01)	**1.04**	**(1.03 to 1.04)**	1.00	(0.99 to 1.01)	1.00	(0.99 to 1.01)
MUAC of mother		0.99	(0.98 to 1.00)	**0.89**	**(0.88 to 0.90)**	**0.98**	**(0.97 to 0.98)**	**0.97**	**(0.94 to 0.99)**
**Food and nutrition**									
Carbohydrate		**0.90**	**(0.87 to 0.93)**	**0.89**	**(0.87 to 0.91)**	**0.95**	**(0.92 to 0.98)**	**0.95**	**(0.92 to 0.98)**
Protein		**0.70**	**(0.68 to 0.72)**	**0.87**	**(0.84 to 0.89)**	**0.94**	**(0.91 to 0.96)**	**0.94**	**(0.91 to 0.97)**
Fats		1.01	(0.81 to 1.25)	**0.96**	**(0.94 to 0.98)**	0.96	(0.79 to 1.17)	**0.95**	**(0.93 to 0.97)**
Fruits and vegetables		0.94	(0.82 to 1.08)	0.94	(0.82 to 1.08)	0.98	(0.95 to 1.01)	0.98	(0.87 to 1.11)
**Climatic/environmental data**									
Season (Deyr as reference)	Gu	**0.85**	**(0.83 to 0.87)**	**0.81**	**(0.79 to 0.83)**	0.80	(0.62 to 1.04)	0.92	(0.85 to 1.01)
	Hagaa	**1.12**	**(1.08 to 1.16)**	**1.11**	**(1.07 to 1.14)**	**1.31**	**(1.25 to 1.37)**	**1.09**	**(1.03 to 1.15)**
	Jilaal	**1.31**	**(1.27 to 1.36)**	**1.33**	**(1.30 to 1.37)**	**1.03**	**(1.01 to 1.06)**	**1.10**	**(1.07 to 1.13)**
	Distance to water	1.00	(1.00 to 1.00)	1.00	(1.00 to 1.00)	1.00	(1.00 to 1.00)	1.00	(1.00 to 1.00)
Enhanced vegetation index (EVI)		**0.42**	**(0.12 to 1.43)**	**0.39**	**(0.35 to 0.45)**	**0.45**	**(0.43 to 0.46)**	**0.40**	**(0.39 to 0.41)**
Rainfall		1.00	(0.99 to 1.01)	**0.83**	**(0.83 to 0.84)**	0.99	(0.98 to 1.00)	**0.86**	**(0.85 to 0.87)**
Temperature		**1.12**	**(1.08 to 1.16)**	**1.12**	**(1.08 to 1.16)**	**1.34**	**(1.30 to 1.38)**	**1.32**	**(1.29 to 1.36)**
Urbanisation		0.98	(0.94 to 1.02)	**0.82**	**(0.79 to 0.86)**	1.02	(0.98 to 1.07)	**0.91**	**(0.89 to 0.93)**
**Interaction terms**									
Carbohydrate: EVI				**1.10**	**(1.02 to 1.19)**			2.54	(0.80 to 8.07)
Carbohydrate: precipitation				1.00	(1.00 to 1.01)			1.00	(1.00 to 1.01)
Protein: EVI				1.22	(0.70 to 2.12)			1.23	(0.34 to 4.41)
Protein: precipitation				1.00	(1.00 to 1.00)			1.00	(1.00 to 1.01)
Recent conflict: carbohydrate				0.96	(0.70 to 1.33)			0.71	(0.35 to 1.43)
Recent conflict: protein				0.67	(0.17 to 2.72)			0.51	(0.21 to 1.25)
Recent conflict: EVI				1.37	(0.69 to 2.71)			2.17	(0.70 to 6.73)
Recent conflict: precipitation				**1.15**	**(1.02 to 1.30)**			**1.22**	**(1.08 to 1.37)**
Longer term conflict: carbohydrate				0.78	(0.30 to 2.00)			0.79	(0.20 to 3.14)
Longer term conflict: protein				0.92	(0.39 to 2.21)			0.80	(0.39 to 1.65)
Longer term conflict: EVI				**1.91**	**(1.79 to 2.04)**			**1.98**	**(1.67 to 2.34)**
Longer term conflict: precipitation				0.98	(0.95 to 1.01)			**0.96**	**(0.93 to 0.99)**
EVI: temperature				**0.19**	**(0.06 to 0.55)**			**0.28**	**(0.09 to 0.90)**

CRI, credible interval; IDP, internally displaced persons; MUAC, mid-upper arm circumference.

Older children were at a lower risk of wasting and higher risk of stunting. Children who had diarrhoea, ARI and fever in the last 2 weeks had a higher risk of wasting (OR=1.38, 95% CrI: 1.16 to 1.64; OR=1.27, 95% CrI: 1.07 to 1.50; OR=1.29, 95% CrI: 1.08 to 1.53) and stunting (OR=1.43, 95% CrI: 1.21 to 1.68; OR=1.12, 95% CrI: 1.08 to 1.16; OR=1.16, 95% CrI: 1.12 to 1.20). Household access to food high in carbohydrates and protein was associated with lower risk of both wasting and stunting.

Change of season from Deyr (October–November short rains) to Gu (April–June long rains) reduced the risk of wasting but was not associated with stunting. The change from Deyr (October–November short rains) to the dry seasons Jilaal (December–March main dry season) and Hagaa (July–September short dry) was associated with higher risk of both wasting and stunting. Vegetation cover was associated with lower risk of wasting and stunting, while increased temperature was associated with higher risk of wasting and stunting.

### Model B: accounting for the effect of conflict

**Figure 1:**
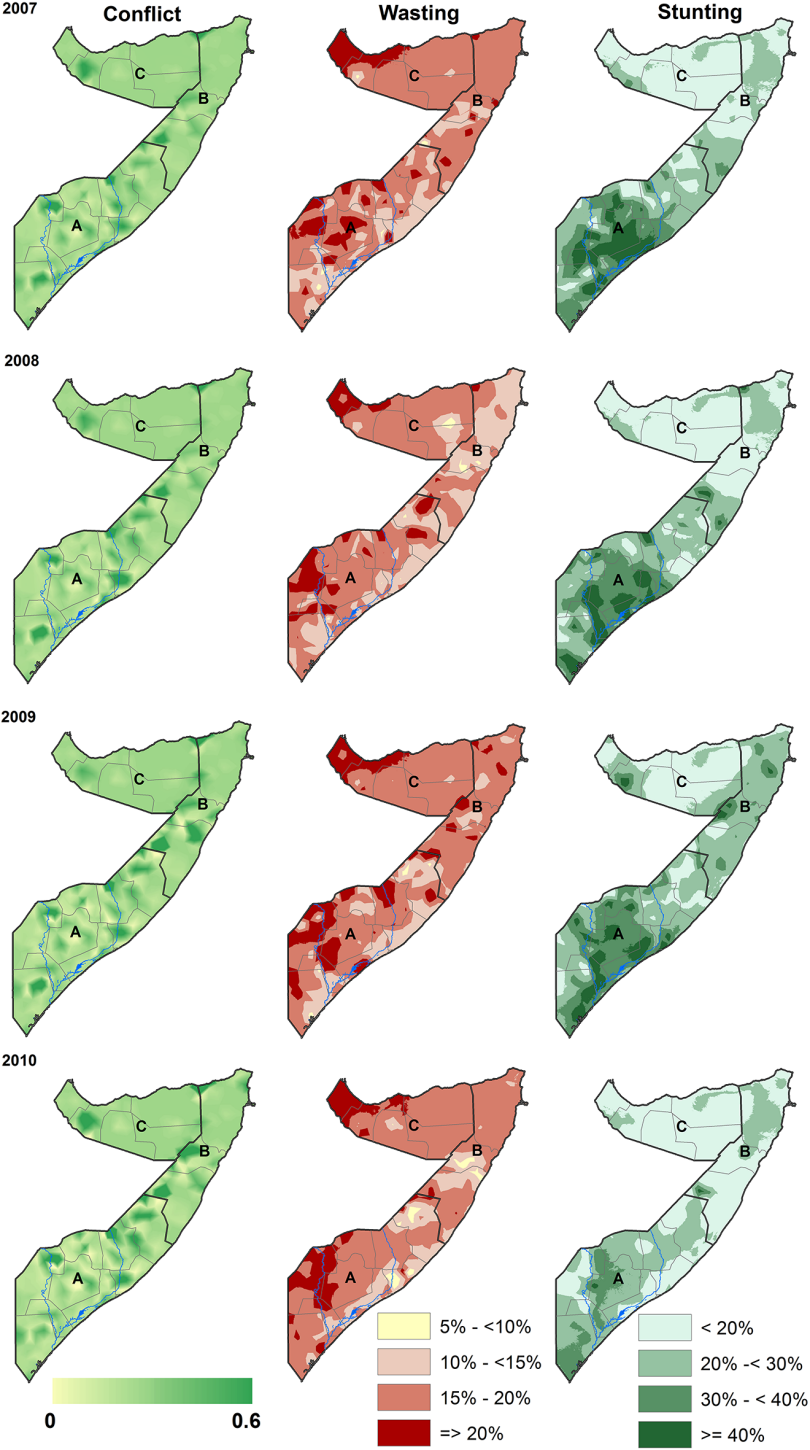
Smooth maps showing the distribution of reported conflict, the prevalence of wasting and the prevalence of stunting between 2007 and 2010 in Somalia. The country is divided into three main zones: South Central (**A**), North East (**B**) and North West (**C**). The country’s two main rivers Juba and Shebelle are located in the South Central zone.

**Figure 2:**
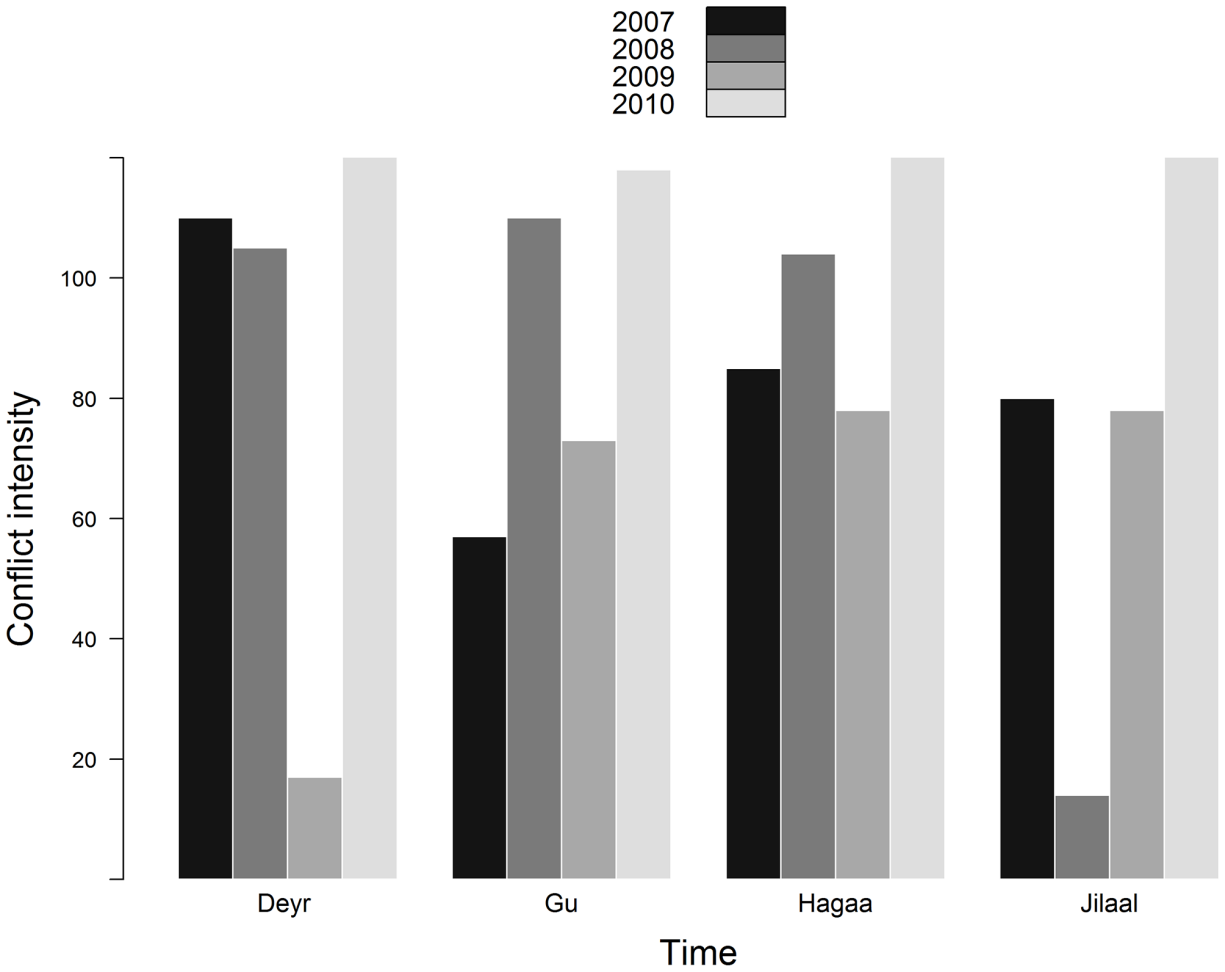
Number of conflict events for the period 2007–2010 grouped in four seasons in Somalia. ‘Deyr’=October–December, ‘Gu’=April–June, ‘Hagaa’=July–September and ‘Jillal’=December–March.

Figure 1 shows the distribution of conflict, wasting and stunting. Out of the 1395 total conflict events recorded from 2007 to 2010, 760 (54%) were battles, 109 (8%) were remote violence, 393 (28%) were violence against civilians and 133 (10%) were in the other types of conflict events. A high number of conflict events occurred in the South Central region. A similar distribution was observed for both wasting and stunting. The seasonal timing of conflict events is shown in figure 2. Monthly fluctuation of conflict events for the period of 2007–2010 is shown in Figure SI.1.

The correlations between covariates were found to be lower than 0.6, and therefore all the predictor variables were used in the main spatial-temporal Bayesian logistic analysis (Figure SI. 2). The effects of the covariates used in the models are shown in table 2. In this model, the recent conflict was associated with wasting (OR=1.37, 95% CrI: 1.33, 1.42) and with stunting (OR=1.21, 95% CrI: 1.15, 1.28). Longer term conflict had larger ORs for wasting (OR=1.76, 95% CrI: 1.71, 1.81) and stunting (OR=1.88, 95% CrI: 1.83, 1.94). The protective associations between rainfall, urbanisation and undernutrition became statistically significant after controlling for conflict. In addition, estimates of the effect size for being IDP increased: OR=1.62, 95% CrI: 1.54, 1.70; OR=2.23, 95% CrI: 2.07, 2.40 for wasting and stunting, respectively.

The interaction terms between carbohydrate and EVI, recent conflict and precipitation, longer term conflict and EVI, and EVI and temperature were significantly associated with the risk of wasting, while recent conflict and precipitation, longer term conflict and EVI, longer term conflict and precipitation, and EVI and temperature are significantly associated with the risk of stunting.

The AFs for recent conflict were 7.6% and 6.9%, while the AFs of longer term conflict were 6.0% and 7.4% for wasting and stunting, respectively. The combined AF of recent and longer term conflicts were 13.1% and 14.4% for wasting and stunting, respectively. Recent conflict and EVI (indicating drought) accounted for 15.4% and 14.4%, while longer term conflict and EVI accounted for 8.6% and 14.5% of wasting and stunting, respectively (table 3). The protective effect of vegetation cover on stunting was enhanced to OR=0.40, 95% CrI: 0.39, 0.41 in model B.

**Table 3:** Attributable fractions (AFs) of recent and longer term conflict and enhanced vegetation index (EVI) for the wasting and stunting among children aged 5–59 months in Somalia

Predictors	Wasting (%)	Stunting (%)
Recent and longer term conflicts	13.12	14.43
Recent conflict and EVI	15.36	14.42
Longer term and EVI	8.60	14.47
Recent conflict	7.62	6.91
Longer term conflict	5.97	7.39
EVI	6.27	6.83

**Figure 3:**
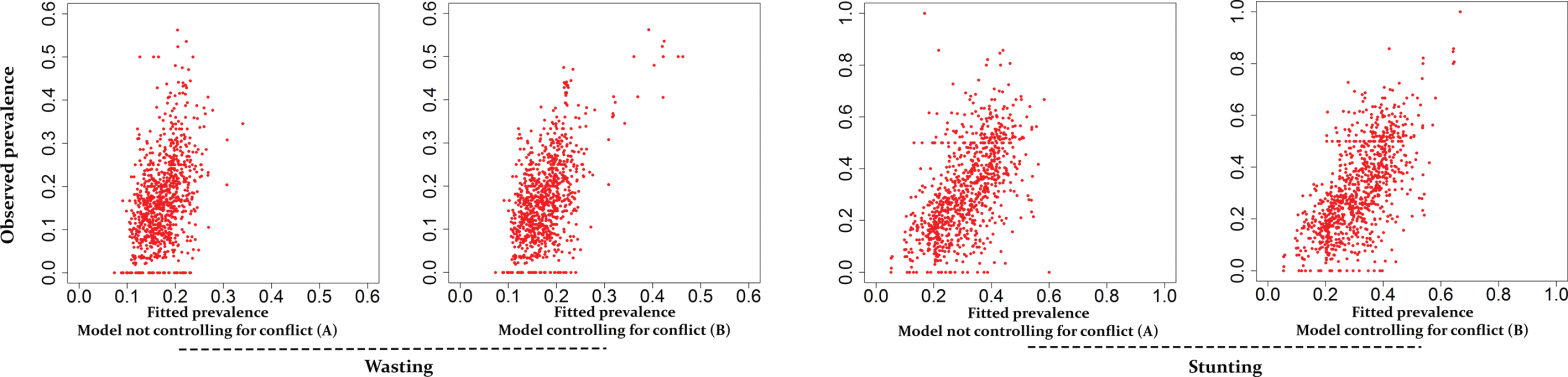
The observed versus fitted prevalence of wasting and stunting from model (**A**) that did not account for the effects of conflict and model (**B**) that accounted for the effects of recent and longer term conflict.

The WAIC value of models improved with the inclusion of conflict: for wasting, from 282.74 to 228.21, and for stunting, from 380.49 to 321.15. The correlation plots between the observed and fitted prevalence of wasting and stunting from both models are shown in figure 3. Adjusted OR of models with any conflict and with recent and longer term conflict are found in Table SI. 3.

## Discussion

The aim of this study was to assess the effect of recorded conflict events on undernutrition among Somali children under 5 years of age. Not surprisingly, conflict negatively impacted children’s nutritional status. However, importantly, the effects of conflict were not fully captured by survey data on individual, household and environmental factors.^6^ The effects of recent and longer term conflict, after adjustment for the individual, household and environmental determinants, were large, with a 76% and 88% greater odds of wasting and stunting and AFs of 13% and 14%, respectively. The main effect of including conflict in the model improved the model fit for high prevalence clusters of both wasting and stunting.

Our findings are consistent with previous studies that suggest that conflict is associated with undernutrition, especially in countries where prolonged conflict overlaps with persistent drought and famine in sub-Saharan Africa.^1^ In South Sudan, nutritional status among children in areas exposed to counterinsurgency warfare was highest among the internally displaced population.^28^ The flight of approximately a million Rwandan refugees into Democratic Republic of Congo in 1994 overwhelmed the international community’s capacity to respond leading to high rates of undernutrition, coupled with serials of epidemics in refugee camps.^29^ The Ethiopian famine coupled with high level of conflict during the late 1990s resulted in migration, undernutrition and high mortality rates, particularly for the marginalised pastoralist communities.^30^

In 2009, approximately 3.25 million people in Somalia were in need of humanitarian assistance of which 1.8 million were children, a 77% increase from early 2008 and around 1 million children were acutely malnourished.^31^ Due to the high level of conflict in the South, which has made access challenging, people continue to migrate both internally and to neighbouring countries.^32^ Over a million people have been internally displaced since fighting resumed in 2006.^31^ Continued crowding, lack of human waste disposal systems and treated public water supplies in displaced person centres and feeding stations have degenerated the living conditions of the Somalis, hence contributing to high levels of undernutrition and diseases.^32^

Poor rainfall and vegetation cover, continued conflict leading to restricted access to humanitarian assistance and internally displacement have been highlighted as the main underlying factors contributing to high rates of undernutrition in Somalia.^32^ A comprehensive analysis of the determinants of undernutrition combined with the effects of conflict for a given population group provide a better basis for action.^2^ Our results suggest that vegetation cover, hence the potential for arable production, becomes more important in the context of conflict. Support for agriculture may, therefore, be beneficial. This study has shown that wasting is sensitive to seasonal variation, with an increase in risk during the dry seasons and a decrease in wet seasons. In addition, female children had a lower risk of both wasting and stunting. These findings are consistent with findings from demographic and health surveys (DHS) in Ethiopia in 2016 and in Kenya in 2014 that showed similar results: girls were at a lower risk compared with boys. This information can be used during emergency humanitarian interventions to target vulnerable children in the dry seasons.^33 34^ In Niger, distribution of ready-to-use-therapeutic foods to non-malnourished children aged 6–59 months was effective in limiting reductions in weight-for-height and reducing the incidence of wasting in the short-term period during the dry seasons.^35^ We found the risk of wasting was highest among children of less than 12 months of age. This again concurs with DHS findings in the region that demonstrate highest prevalence of wasting among infants. Programmes should therefore strengthen capacity to target interventions towards infants, seasonally, and considering recent conflict or a history of conflict locally.^36^

Our study has some limitations. The data were secondary, obtained from several cross sectional surveys conducted by FSNAU and hence children in IDP camps could not be linked to their location of origin where the conflict occurred but were linked to the conflict data in their locations at the time of the surveys. In addition, wasting may vary within a short period, and this variability might not be fully captured from the cross-sectional studies that were intermittent and could not be conducted during periods of active local conflict. Importantly, the surveys, like many others, omitted infants under 6 months old, which have recently been highlighted as having a significant burden of severe acute undernutrition and more likely to have poor outcomes than older children.^36^ The ACLED database may not have captured all conflict events, especially smaller armed violence events.

## Conclusion

Our study shows that the effects of conflict on undernutrition were not fully captured by analysis of the individual, household and environmental variables that we assessed, even though these may be expected to be affected by conflict. These results highlight the necessity to consider conflict in addition to other factors to estimate risk. Mitigating the effects of conflict on undernutrition will require improved analytical and operational framework in order to protect livelihoods and safeguard developmental gains.
