# Supplementary material for: Conflict in Somalia: impact on child undernutrition
Source: BMJ Glob Health. 2017 May 29;2(2):e000262. doi: 10.1136/bmjgh-2016-000262 (PMC5621625; doi:10.1136/bmjgh-2016-000262)
Supplement: Supplementary data [file bmjgh-2016-000262supp001.pdf]

Conflict in Somalia – impact on child malnutrition

Supplementary Information: Model procedures, Data and model output

SI.1 History of conflict in Somalia

Somalia gained independence in 1960 and for the first ten years, the country experienced no significant armed conflict.[1] Somalia has subsequently experienced protracted instability and armed conflict.[2] In 1969, the military seized power in a coup and the Barre regime billed this as a cold war socialist uprising.[1] Before 1990, the country endured three major armed conflicts: the Ogaden war with Ethiopia in 1977-78; the war between the Somali military and the Somali National Movement (SNM) for the control over northwest Somalia; and armed conflict between government forces against growing numbers of clan-based liberation movements in 1989 and 1990. International donors stopped aid to Somalia in response to the war with the SNM, which eventually led to the collapse of the government in 1991. This resulted in destruction of social and economic infrastructure, massive internal and external migration, degradation of the environment, and thus profoundly altered human development.[3] The subsequent absence of a central government led to the emergence of self-declared states of North West "Republic of Somaliland" in 1991, of a Northeastern "Puntland State of Somalia" in 1998, and military administration in the southern regions of Bay and Bakool in 1999. A Transitional Federal Charter of the Somali Republic was adopted in 2004 and although a government was formed, the political and security situation remained unstable.[4] In 2012, a Federal government of Somalia was formed under a constitution following the end of the interim mandate of the Transitional Federal Government (TFG).

Violence escalated from 2006 in response to deployment of African Union (AU) peacekeepers in Mogadishu and the reinforcement of peace by the TFG and Ethiopian government.[1] In 2007, the fighting between TFG and the insurgency resulted in the displacement of approximately 700,000 people from Mogadishu. This was followed by high levels of conflict as a result of military occupation, violent insurgency and eventually massive population displacement which reversed the incremental political and economic progress that had been achieved.[1]

Survey design

For nutrition survey, sample size was calculated in the number of children aged 6-59 months using Epi Info/Ena 2008 software (Center for Disease Control (CDC) in USA) with household as the sampling unit. Therefore the final sample size was converted in terms of number of households.[5 6] This required an estimate

of the average household size and the proportion of children aged 6-59 months in the population which was estimated from a previous survey or from national statistics.

In each survey, a stratified multi-stage cluster sampling design was adopted where the sampling frame of a selected district was based on the four livelihood definitions (pastoral, agro-pastoral, riverine and fishing) within which 30 communities and 30 households within each community were selected at random.[7]

Respective samples sizes (number of households and number of children) were calculated using the Epi Info/Ena 2008 software (Center for Disease Control (CDC) in USA) after considering the population size, estimated prevalence and desired precision. A list of all villages and population within each of the assessed livelihoods served as a sampling frame and was used to construct cumulative population for the assessment area. Selection of respondents within the village was done randomly, preferably from a list of eligible names or a map of households. Where these were not available, the number of households in the village was estimated from the population figures (the total population divided by the mean household size).[8] Further detailed information on FSNAU surveys is available online (http://www.fsnau.org/).[8 9]

SI.2 Spatial-temporal binomial regression model

Specifically, we let y_i to be a binary response variable where $y_i = 1$ if a child is malnourshed and $y_i = 0$ if a child is not malnourished where $i; i = 1, \ldots, 73778.$ We assumed that this number was Binomial with a cluster level prevalence p_i , the response variable is distributed as a Bernoulli random variable such that;

$$f(\mathrm{y}_i \mid \mathrm{n}_i) = \mathrm{p}_i^{y_i}(1-p_i)^{1-y_i} = \exp[\mathrm{y}_i\mathrm{n}_i - \log(1+\exp(\mathrm{n}_i))] \qquad \text{(Equation SI2.1)}$$

where $p_i = P(\mathrm{y}_i = 1),$ and $n_i = \log it(\mathrm{p}_i)$ is a canonical parameter linked to the linear predictor. We modeled the relationship between malnutrition and covariates using a Bayesian hierarichical logistic regression model that account for excess heterogeneity and spatial similarity between clusters. This model is expressed as;

$$\operatorname{logit}(p_i) = \boldsymbol{X}_i^{'}\beta + u_i + v_i \, , \qquad \text{(Equation SI2.2)}$$

where β is a $(p \times 1)$ vector of parameters, $\boldsymbol{X}_i^{'}$ is the covariate matrix, u_i is a spatial structured random effect for cluster i, v_i is an unstructured random effect for cluster i. To model spatial dependence between the random effects u_i in neighbouring areas we assumed a conditional autoregressive (CAR) prior for these terms.[10] Each u_i was modelled (conditional on its weighted neighbors) as normally distributed with mean equal to the mean of the effects of its neighbors and a variance that is inversely proportional to the number of neighbours N_i, that is, $u_i \mid \mathbf{u}_{-i} \sim N\left(\mu_i, \sigma_i^2\right)$, where $\mathbf{u}_{-i}$ is a vector of all the spatial random effects except for the one at the i-th cluster and $\mu_i = \frac{\phi}{n_i} \sum_{j \in N_i} u_j$ and $\sigma_i^2 = \frac{1}{\tau^2 n_i}$, where n_i is the number of clusters in the neihbourhood of the i-th cluster, and τ is the precision parameter. The joint distribution of the v_i's is an independent multivariate normal. In this approach, the two models were implemented using Integrated Nested Laplace Approximation (INLA) as implemented in R-INLA in R project version 3.2.3.[11]

Table SI. 1: Description of the ACLED conflict by event type categories used in this study. ACLED codes for nine types of events: battle – no change of territory, battle – non-state actor overtakes territory, battle – government regains territory, headquarters or base established, strategic development, riots or protests, violence against civilians, non-violent transfer of territory and remote violence.[12 13] These event types were further grouped into four classes for the analysis: battle, remote violence, violence against civilians and others.

Study class	ACLED Class	Event description
**Battle**	This is a violent interaction between two politically organized armed groups at a particular time and location.	
	Battle-No change of territory	A battle between two armed groups where control of the contested location does not change.
	Battle-Non-state actor overtakes territory	A battle between two armed groups where non-state actor win control of the contested location.
	Battle-Government regains territory	A battle in which the government regains control of a location.
**Remote violence**	This is where one group is spatially removed but perpetrator did not require physical presence.	
	Remote violence	Refers to an event in which the means for engaging in conflict did not require the physical presence on the perpetrator. This include bombings, mortar and missile attacks
**Violence against civilians**	This is a deliberate violent attacks perpetrated by an organized political group such as a rebel, militia or government force against unarmed non-combatants.	
	Violence against civilians	This occurs when any armed or violent group attacks civilians. This include rebels, militias and rioters among others.
	Riots/Protests	This records group public demonstration, often against a government institution.
**Other**	This category capture non-violent events that may trigger future events.	
	Headquarters or base established	This is a non-violent event where non-state group establishes a base or headquarters.
	Strategic development	This is a non-violent event but within the context of the war/dispute that records activity by rebel groups/militia/governments. For instance location and date of peace talks and arrests of high-ranking officials.
	Non-violent transfer of territory	This event describes situations in which rebels or governments acquire control of a location without engaging in a violent act.

Table SI 2: Summary of survey data aggregated for the period 2007-2010 (FSNAU 2007-2010). Values in parentheses, next to the number of children, are percentages.

**Characteristic**	**Number**			
Total number of children examined	73778			
Total number of clusters examined	1066			
**Summary by livelihood**	**Wasting; n=15735, (%)**		**Stunting; n=22739 (%)**	
Livelihood Agro-pastoral	4406(28)		7276(32)	
Pastoral	4878 (31)		5230 (23)	
Riverine	2990 (19)		5685 (25)	
Urban areas	787 (5)		910 (4)	
Internally Displaced Persons	2518 (16)		3638 (16)	
	**Wasted; n = 15735 (21)**	**Not wasted; n=58043 (79)**	**Stunted; n =22739 (31)**	**Not stunted ; n =51039 (69)**
**Child data**	**Number (%)**		**Number (%)**	
Vitamin A supplementation	8995 (57)	33356 (57)	12853 (57)	29498 (58)
Measles vaccination	8367 (53)	30875 (53)	11919 (52)	27317 (54)
Polio vaccination	12346 (78)	46519 (80)	18272(80)	40647 (80)
Diarrhoea in the last 2 weeks	3749 (24)	10674 (18)	5136 (23)	9159 (18)
Acute Respiratory Infection	3797 (24)	12029(21)	5090 (22)	10647 (21)
Febrile Illness in the last 2 weeks	3793 (24)	11912 (21)	4849 (21)	10753 (21)
Suspected measles in last 1 month	788 (5),	2487 (4)	1026 (5)	2229 (4)
Sex of the child	Male=9039 (57)	Male= 28761 (50)	Male=12776 (56)	Male= 24844 (49)
Age of the child (months)	Mean=33, Range=(6,59)	Mean = 33, Range=(6,59)	Mean =31, Range=(6,59)	Mean= 32, Range=(6,59)
Age of the mother (years)	Mean=30, Range = (15,60)	Mean = 30, Range = (15,60)	Mean=30, Range = (15,60)	Mean = 30, Range = (15,60)
MUAC of mother cm	Mean =21, Range=(18,38)	Mean = 22, Range=(18,38)	Mean=22, Range=(18,38)	Mean= 22, Range=(18,38)
**Food access data**	**Number (%)**		**Number (%)**	
High carbohydrate foods in the last 24 hours	15227 (97)	56059 (97)	22285(98)	49008 (96)
High protein foods in the last 24 hours	13420 (85)	50543 (87)	19691 (87),	44322 (87)
Fats in the last 24 hours	12327 (78)	46277(80)	17751 (78),	40880 (80)
Fruits and vegetables in the last 24 hours	6423(41)	24835 (43)	10695 (47)	20665 (40)
**Household data**	**Mean (Range)**		**Mean (Range)**	

Household size	6 (2, 50)	6 (2, 50)
Number of under 5s	2 (1, 5)	2 (1, 5)
Household head gender	Male=60128 (81%)	Male=60128 (81%)
**Cluster data**	**Mean (Range)**	**Mean (Range)**
Distance to water to major water bodies (km)	97 (0,508)	97 (0,508)
Enhanced Vegetation Index (EVI)	0·18 (0,0·45)	0·18 (0,0·45)
Precipitation (mm/year)	138 (0,350)	138 (0,350)
Mean Temperature (˚C)	28 (21,31)	28 (21,31)
Urbanization	Urban = 3318 (5%), Rural = 70460	Urban = 3318 (5%), Rural = 70460

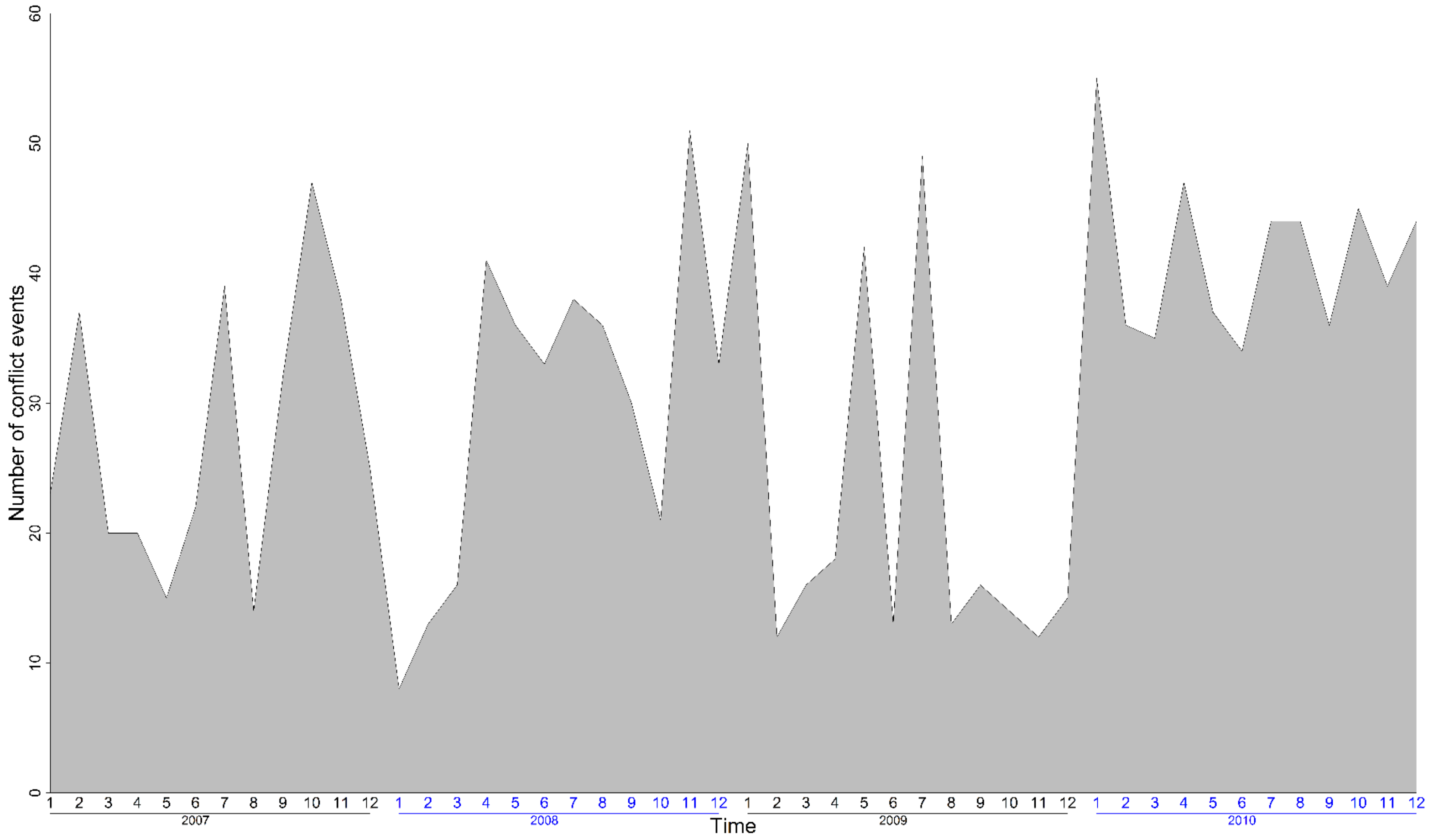

Figure SI. 1: Monthly fluctuation of conflict events for the period of 2007 – 2010.

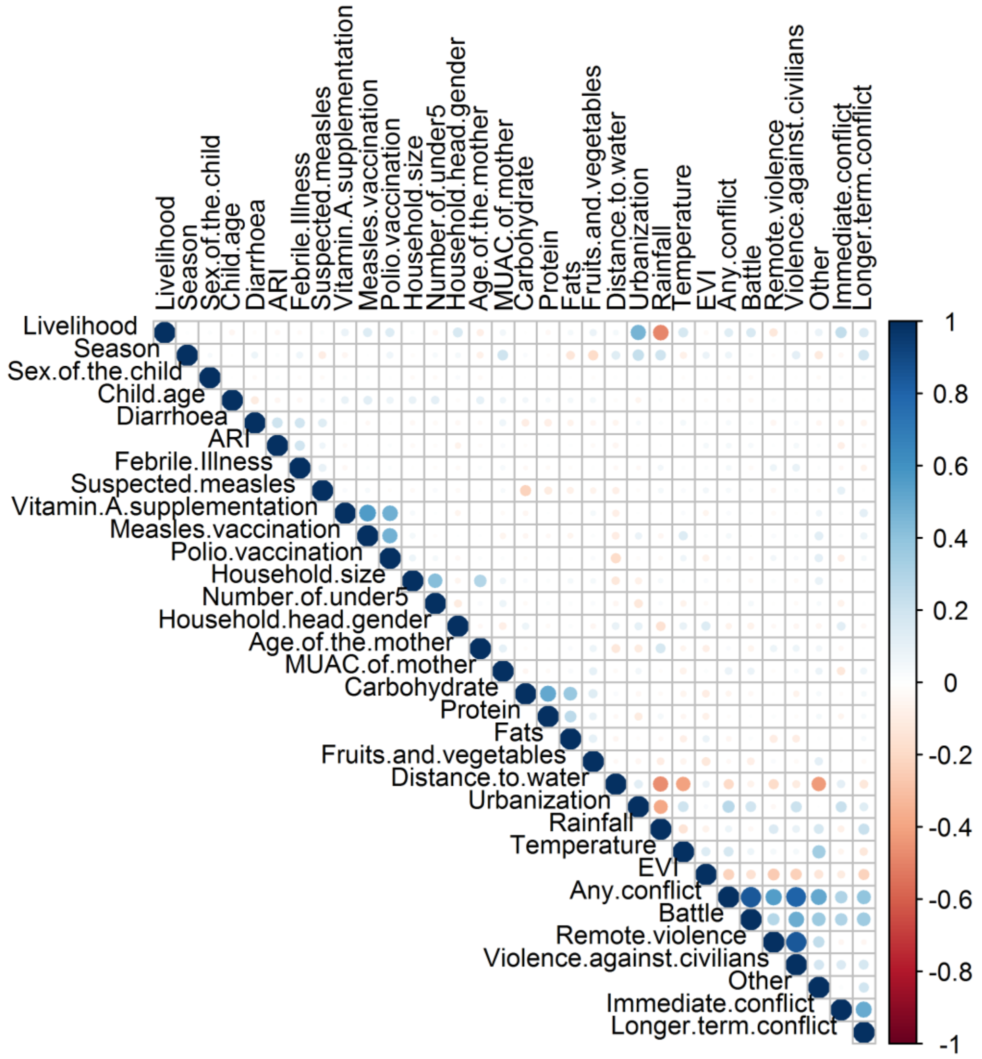

Figure SI. 2: Correlation between covariates used in the spatial-temporal Bayesian logistic analysis.

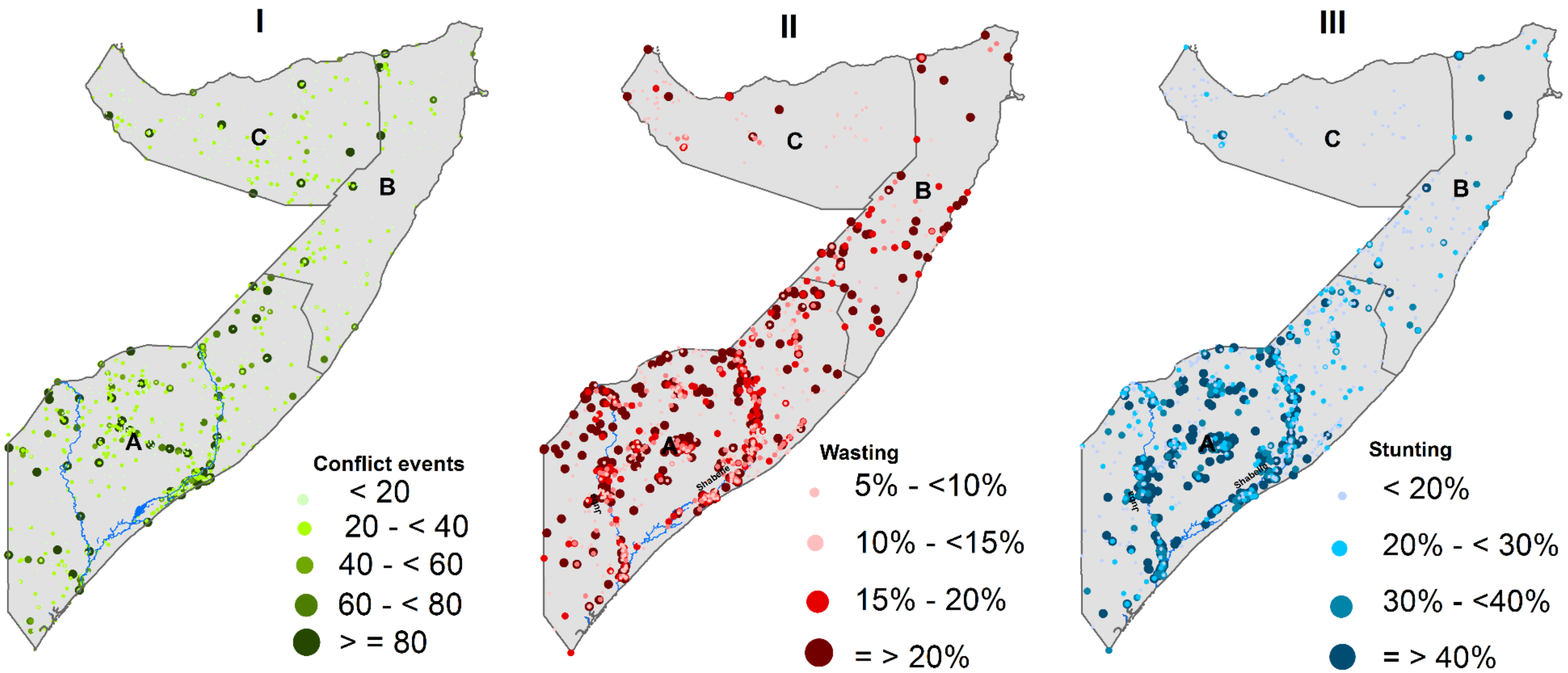

Figure SI. 3: Maps showing the location of nutrition survey clusters and (I), the number of reported conflict, (II) the observed prevalence of wasting (III) and the observed prevalence of stunting between 2007 and 2010 in Somalia. The country is divided into three main zones: South Central (A), North East (B) and North West (C). The country's two main rivers, Juba and Shebelle are located in the South Central zone.

Table SI. 3: Adjusted odds ratio (POR) and 95% credible interval (CrI) of the effect of covariates on wasting and stunting among children aged 6 – 59 months in Somalia. The estimates were derived from three models by type of event: Model that did not account for the effect of conflict as covariate; Model that accounted for the effect of any conflict and Model that accounted for the effect of recent and longer term conflict. Values in bold typeface do not contain the value 1 in their 95% CrI, and were considered statistically significant.

		**Wasting**						**Stunting**					
		**Model without conflict**		**Model with Any conflict**		**Model with Recent and Longer term conflict**		**Model without conflict**		**Model with Any conflict**		**Model with Recent and Longer term conflict**	
**Correlates**		**Odds ratio**	**CrI**	**Odds ratio**	**CrI**	**Odds ratio**	**CrI**	**Odds ratio**	**CrI**	**Odds ratio**	**CrI**	**Odds ratio**	**CrI**
**Conflict data**													
Any battle				**1·43**	**(1·38,1·49)**					**2·55**	**(2·41,2·70)**		
Any remote violence				**1·49**	**(1·42,1·56)**					**2·00**	**(1·89,2·12)**		
Any violence against civilians				**1·54**	**(1·53,1·55)**					**1·86**	**(1·76,1·97)**		
Any other				**1·25**	**(1·19,1·31)**					**1·82**	**(1·65,2·00)**		
Recent (within 3 months)conflict	Battle					**1·34**	**(1·30,1·39)**					**1·32**	**(1·28,1·36)**
	Remote violence					**1·20**	**(1·17,1·23)**					**1·20**	**(1·15,1·26)**
	Violence against civilians					**1·21**	**(1·18,1·24)**					**1·17**	**(1·13,1·21)**
	Other					**1·19**	**(1·14,1·24)**					**1·18**	**(1·14,1·23)**
Longer term conflict (3 to 12 months ago)	Battle					**1·28**	**(1·25,1·32)**					**1·44**	**(1·40,1·47)**
	Remote violence					**1·19**	**(1·13,1·25)**					**1·28**	**(1·22,1·34)**
	Violence against civilians					**1·24**	**(1·19,1·29)**					**1·22**	**(1·18,1·26)**
	Other					**1·24**	**(1·19,1·29)**					**1·33**	**(1·28,1·38)**
**Livelihood** (Agro-pastoral as reference)	Pastoral	**0·94**	**(0·91,0·97)**	**0·91**	**(0·89,0·94)**	**0·82**	**(0·80,0·85)**	**0·60**	**(0·41,0·87)**	**0·71**	**(0·68,0·74)**	**0·66**	**(0·61,0·71)**
	Riverine	**1·13**	**(1·09,1·17)**	**1·18**	**(1·14,1·23)**	**1·18**	**(1·16,1·21)**	**1·44**	**(1·04,1·99)**	**1·40**	**(1·35,1·45)**	**1·51**	**(1·47,1·55)**
	Urban	0·98	(0·94,1·02)	1·02	(0·58,1·80)	**1·18**	**(1·15,1·21)**	0·98	(0·93,1·04)	**0·83**	**(0·78,0·88)**	**0·88**	**(0·86,0·90)**
	IDP	**1·37**	**(1·34,1·41)**	**1·67**	**(1·06,2·61)**	**1·25**	**(1·21,1·29)**	**1·91**	**(1·05,3·48)**	**1·84**	**(1·73,1·95)**	**1·96**	**(1·91,2·02)**
**Child data**													

Vitamin A supplementation		**0·82 (0·81,0·84)**	**0·88(0·85,0·91)**	**0·84 (0·82,0·86)**	1·10 (0·92,1·31)	0·91 (0·76,1·08)	0·91 (0·71,1·17)
Measles vaccination		0·87 (0·71,1·05)	**0·86(0·83,0·89)**	**0·89 (0·85,0·92)**	0·87 (0·73,1·04)	**0·88 (0·85,0·91)**	**0·91 (0·86,0·96)**
Polio vaccination		**0·89 (0·87,0·91)**	**0·93(0·91,0·95)**	**0·83 (0·77,0·89)**	**0·91 (0·87,0·94)**	**0·95 (0·93,0·97)**	**1·04 (1·02,1·07)**
Diarrhoea		**1·38 (1·16,1·64)**	**1·39(1·17,1·66)**	**1·35 (1·28,1·44)**	**1·43 (1·21,1·68)**	**1·43 (1·22,1·68)**	**1·29 (1·22,1·36)**
Acute Respiratory Infection (ARI)		**1·27 (1·07,1·50)**	**1·27(1·07,1·50)**	**1·21 (1·14,1·29)**	**1·12 (1·08,1·16)**	**1·13 (1·09,1·17)**	**1·21 (1·15,1·28)**
Febrile Illness		**1·29 (1·08,1·53)**	**1·30(1·09,1·54)**	**1·27 (1·24,1·30)**	**1·16 (1·12,1·20)**	**1·16 (1·12,1·21)**	**1·17 (1·14,1·20)**
Suspected measles		1·13 (0·98,1·32)	0·88(0·65,1·20)	1·05 (0·94,1·18)	1·02 (0·99,1·04)	1·05 (0·80,1·37)	1·01 (0·98,1·04)
Sex of the child (female)		**0·72 (0·63,0·83)**	**0·72(0·63,0·83)**	**0·73 (0·69,0·76)**	**0·75 (0·66,0·86)**	**0·75 (0·66,0·86)**	**0·73 (0·70,0·76)**
Chid age (< 12 reference)	12 - < 24 months	**0·57 (0·53,0·61)**	**0·56(0·53,0·60)**	**0·66 (0·65,0·68)**	**3·36 (3·27,3·46)**	**3·32 (2·50,4·42)**	**2·34 (2·23,2·46)**
	24 – 59 months	**0·69 (0·55,0·87)**	**0·68(0·54,0·86)**	**0·50 (0·46,0·54)**	**2·34 (2·28,2·41)**	**2·31 (1·77,3·03)**	**2·05 (1·89,2·23)**
**Household data**							
Household size		**1·04 (1·01,1·08)**	**1·10(1·06,1·14)**	**1·10 (1·08,1·11)**	**1·04 (1·01,1·08)**	**1·04 (1·00,1·07)**	**1·09 (1·08,1·10)**
Number of under 5s		**1·07 (1·02,1·13)**	**1·09(1·06,1·12)**	**1·07 (1·04,1·11)**	**1·04 (1·02,1·07)**	**1·04 (1·01,1·07)**	**1·06 (1·03,1·08)**
Female household head		1·06 (0·89,1·28)	1·06(0·88,1·27)	**1·04 (0·97,1·11)**	**1·22 (1·02,1·45)**	**1·24 (1·04,1·47)**	**1·21 (1·14,1·28)**
Age of the mother (years)		1·00 (0·99,1·01)	**1·07(1·05,1·08)**	**1·14 (1·13,1·14)**	1·00 (0·99,1·01)	1·00 (0·99,1·01)	0·98 (0·95,1·01)
MUAC of mother cm		0·99 (0·98,1·00)	**0·88(0·87,0·89)**	**0·92 (0·92,0·92)**	**0·98 (0·97,0·98)**	**0·98 (0·97,0·99)**	**0·92 (0·92,0·93)**
**Food and nutrition**							
Carbohydrate		**0·90 (0·87,0·93)**	**0·89(0·86,0·92)**	**0·89 (0·84,0·93)**	**0·95 (0·92,0·98)**	**0·95 (0·91,0·98)**	**0·94 (0·91,0·96)**
Protein		**0·70 (0·68,0·72)**	**0·86(0·79,0·95)**	**0·76 (0·74,0·78)**	**0·94 (0·91,0·96)**	**0·93 (0·90,0·95)**	**0·92 (0·90,0·95)**
Fats		1·01 (0·81,1·25)	**0·88(0·86,0·90)**	**0·85 (0·80,0·91)**	0·96 (0·79,1·17)	**0·95 (0·92,0·99)**	**0·91 (0·86,0·97)**
Fruits and vegetables		0·94 (0·82,1·08)	0·93(0·82,1·07)	0·92 (0·80,1·05)	0·98 (0·95,1·01)	0·99 (0·88,1·12)	0·99 (0·96,1·02)
**Climatic / Environmental data**							
Season (Deyr as reference)	Gu	**0·85 (0·83,0·87)**	**0·85(0·83,0·87)**	**0·85 (0·80,0·90)**	0·80 (0·62,1·04)	0·88 (0·74,1·06)	0·92 (0·73,1·14)
	Hagaa	**1·12 (1·08,1·16)**	**1·14(1·10,1·18)**	**1·12 (1·08,1·16)**	**1·31 (1·25,1·37)**	**1·13 (1·08,1·18)**	**1·10 (1·07,1·14)**
	Jilaal	**1·31 (1·27,1·36)**	**1·45(1·37,1·54)**	**1·25 (1·21,1·30)**	**1·03 (1·01,1·06)**	**1·13 (1·05,1·21)**	**1·20 (1·03,1·40)**
Distance to water		1·00 (1·00,1·00)	1·00(1·00,1·00)	1·00 (0·88,1·14)	1·00 (1·00,1·00)	1·00 (1·00,1·00)	1·00 (1·00,1·00)
Enhanced Vegetation Index (EVI)		**0·42 (0·12,1·43)**	**0·36(0·32,0·41)**	**0·54 (0·52,0·56)**	**0·45 (0·43,0·46)**	**0·64 (0·57,0·73)**	**0·49 (0·48,0·51)**
Rainfall		1·00 (0·99,1·01)	**0·82(0·82,0·83)**	**0·84 (0·81,0·88)**	0·99 (0·98,1·00)	**0·88 (0·88,0·89)**	**0·86 (0·84,0·88)**
Temperature		**1·12 (1·08,1·16)**	**1·16(1·12,1·20)**	**1·14 (1·10,1·18)**	**1·34 (1·30,1·38)**	**1·17 (1·13,1·20)**	**1·21 (1·17,1·26)**
Urbanization		0·98 (0·94,1·02)	**0·91(0·87,0·95)**	**0·86 (0·83,0·90)**	1·02 (0·98,1·07)	**0·91 (0·88,0·93)**	**0·84 (0·81,0·87)**

References

1. Conciliation-resources. Endless war: a brief history of the Somali conflict. http://wwwc-rorg/downloads/Accord%2021_3Endless%20war_a%20brief%20history_2010_ENGpdf Accessed 16 May 2016.
2. Bank W. Conflict in Somalia: Drivers and Dynamics http://siteresourcesworldbankorg/INTSOMALIA/Resources/conflictinsomaliapdf Accessed 18 May 2016.
3. UNFAO. Nutrition Country Profile Somalia ftp://ftpfaoorg/es/esn/nutrition/ncp/sompdf 2005.
4. WHO. Somalia Nutrition Strategy 2011 - 2013. http://reliefwebint/sites/reliefwebint/files/resources/Full_Report_1768pdf 2010.
5. Food, of the United Nations/Food Security AO, Unit NA. Guidelines for Emergency Nutrition \& Mortality Surveys in Somalia, 2011.
6. Smith E. Nutritional Surveillance Systems in Somalia, 2006.
7. Noor AM, Clements ACA, Gething PW, et al. Spatial prediction of Plasmodium falciparum prevalence in Somalia. Malaria journal 2008;**7**(1).
8. UN-FAO/FSNAU-Somalia. Food Security and Nutrition Analysis Post Deyr 2010/11, 2011. http://www.fao.org/3/a-as796e.pdf.
9. UN-FAO/FSNAU-Somalia. FSNAU Nutrition Update, May-June 2011. 2011. http://www.fao.org/docrep/014/i2330e/i2330e.pdf.
10. Besag J, York J, Mollié A. Bayesian image restoration, with two applications in spatial statistics. Annals of the Institute of Statistical Mathematics 1991;**43**(1):1-20.
11. Rue H, Martino S, Chopin N. Approximate Bayesian inference for latent Gaussian models by using integrated nested Laplace approximations. Journal of the royal statistical society: Series b (statistical methodology) 2009;**71**(2):319-92.
12. ACLED. Armed Conflict Location & Event Data Project (ACLED) User Guide. http://wwwacleddatacom/wp-content/uploads/2016/01/ACLED_User-Guide_2016pdf 2016.
13. ACLED. Armed Conflict Location and Event Data Project (ACLED) Codebook. http://wwwacleddatacom/wp-content/uploads/2016/01/ACLED_Codebook_2016pdf Accessed 18 May 2016 2016.
